# Recent Perspectives on Anticancer Potential of Coumarin Against Different Human Malignancies: An Updated Review

**DOI:** 10.1002/fsn3.4696

**Published:** 2024-12-31

**Authors:** Muhammad Shahbaz, Asfa Perween, Ushna Momal, Muhammad Imran, Muhammad Hammad Ul Hassan, Hammad Naeem, Ahmed Mujtaba, Muzzamal Hussain, Suliman A. Alsagaby, Waleed Al Abdulmonem, Mohamed A. Abdelgawad, Ahmed H. El‐Ghorab, Samy Selim, Ehab M. Mostafa, Entessar Al Jbawi

**Affiliations:** ^1^ Department of Food Science and Technology Muhammad Nawaz Shareef University of Agriculture Multan Multan Pakistan; ^2^ Department of Human Nutrition and Dietetics Muhammad Nawaz Shareef University of Agriculture Multan Multan Pakistan; ^3^ Department of Food Science and Technology University of Narrowal Narrowal Pakistan; ^4^ Post Harvest Research Centre Ayub Agricultural Research Institute Faisalabad Faisalabad Pakistan; ^5^ Department of Food Science and Technology, Faculty of Engineering Sciences and Technology Hamdard University Islamabad Campus Islamabad Pakistan; ^6^ Department of Food Sciences Government College University Faisalabad Faisalabad Pakistan; ^7^ Department of Medical Laboratory Sciences, College of Applied Medical Sciences Majmaah University Al‐Majmaah Saudi Arabia; ^8^ Department of Pathology, College of Medicine Qassim University Buraidah Saudi Arabia; ^9^ Department of Pharmaceutical Chemistry, College of Pharmacy Jouf University Sakaka Aljouf Saudi Arabia; ^10^ Department of Chemistry, College of Science Jouf University Sakaka Saudi Arabia; ^11^ Department of Clinical Laboratory Sciences, College of Applied Medical Sciences Jouf University Sakaka Saudi Arabia; ^12^ Department of Pharmacognosy, College of Pharmacy Jouf University Sakaka Saudi Arabia; ^13^ Pharmacognosy and Medicinal Plants Department, Faculty of Pharmacy (Boys) Al‐Azhar University Cairo Egypt; ^14^ Agricultural Extension Directorate, MAAR Damascus Syria

**Keywords:** angiogenesis, anti‐apoptotic protein, anticancer mechanisms, apoptosis, blood vessels, efficacy, esculetin, proliferation, T lymphocytes

## Abstract

Coumarins, a group of naturally occurring compounds, have been reported to demonstrate anticancer potential. These substances, distinguished by their combined benzene and α‐pyrone rings, have been demonstrated to impact multiple cellular mechanisms essential for the initiation and advancement of cancer. These agents work in different ways that prevent different tumor cells from growing, spreading, and increasing. One of the main anticancer mechanisms of coumarin act is killing cancer cells through apoptosis. This includes changes to pro‐ and anti‐apoptotic proteins like Bcl‐2 and Bax, the release of cytochrome c from mitochondria, and the activation of caspases. The tumor suppressor protein p53's expression has been discovered to be upregulated by coumarins such as esculetin and imperatorin, which encourage interrupted cell cycle and death. Additionally, coumarin has anti‐angiogenic qualities, which are critical for the development and spread of tumors. It can slow the development of new blood vessels that feed tumors by inhibiting the “vascular endothelial growth factor (VEGF)” route of signaling. Coumarins inhibit the number of signaling pathways that are vital for cell division. For example, they can suppress the “PI3K/mTOR” pathway, which usually impairs the cancer cells and results in decreased cell viability and growth. Finally, coumarins could modulate the response of the immune system to cancerous cells. They have the ability to boost the activity of natural killer cells and cytotoxic T lymphocytes, which aid the immune system in identifying and eliminating cancer cells. Through a variety of mechanisms, such as immune response regulation, angiogenesis reduction, cell growth inhibition, and apoptosis activation, coumarins exhibit their anticancer effects. These molecular pathways demonstrate coumarins' potential as an interesting option for the development of novel anticancer treatments. More studies are needed to completely understand their modes of action and maximize their therapeutic efficacy.

## Graphical abstract

The paper highlights coumarin's ability to induce apoptosis, inhibit cell proliferation, and interfere with cancer cell signaling pathways. Coumarin disrupts key signaling pathways, including NF‐κB and PI3K/Akt, which are crucial for cancer cell survival and proliferation. The paper discusses its impact on various cancers, including breast, lung, and colon cancers, showing its potential as a complementary therapeutic agent. Its low toxicity profile further underscores its potential as a safe adjunct therapy, supporting further research into clinical applications for cancer treatment.

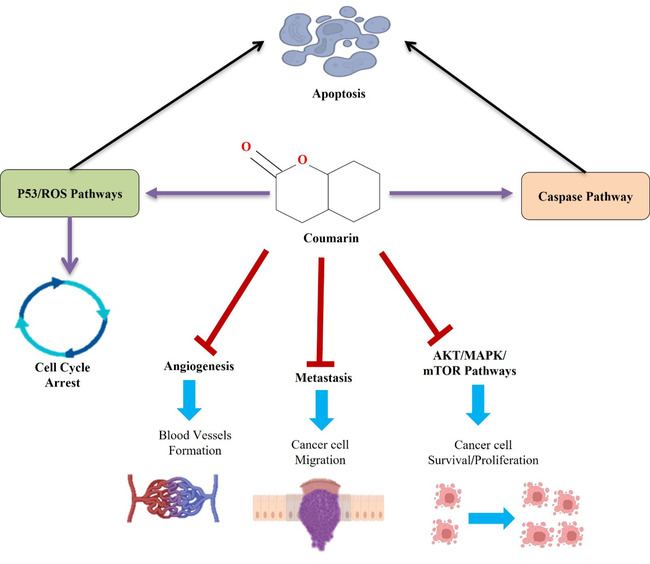

## 1 Introduction

Coumarins are a class of oxygenated, colorless, crystalline, heterocyclic compounds that are rich in polyphenols. These are primarily identified from the plant *Dipteryx odorata* Wild. The Fabaceae family is known locally as “Coumaroun.” Natural substances such as coumarin (2H‐1‐benzopyran‐2‐one or 1, 2‐benzopyrone) and derivatives of coumarin are abundantly found in plants in both free and heteroside form. Around 600 genera and 100 families have yielded 800 naturally occurring coumarin derivate chemicals. In the Dicotyledonae class of the division of spermatophytes, many plant species, especially those belonging to the Rutaceae and Apiaceae families, are frequently discovered to have coumarin and its derivative in their seeds, roots, and leaves. Novobiocin is one of the coumarins that are derived from vascular plants (Akkol et al. 2020).

A benzene ring fused with an alpha‐pyrone ring makes up the fundamental structure of coumarin and its derivatives. Methyl group and hydroxide radicals are catalyzed in diverse strategies for synthesizing coumarin such as 4‐hydroxycoumarin, umbelliferone, esculetin, and scopoletin. Alternatively, complex coumarin originating from phenylpropanoid routes falls into three categories: pyrano‐coumarins, furanocoumarins, and pyrone‐substituted coumarins (Zou et al. 2024).

Naturally, in all members of the plant kingdom, coumarins have a pleasant and aromatic quality. The plant sources of coumarin include fruits and vegetables, spices, and herbs. It is also present in a wide variety of other plants, including tonka beans, woodruff, bison grass, sweet clover, vanilla grass, cinnamon, apricot, cherry, black currant, and strawberry (Lončarić et al. 2020). The coumarins demonstrate a wide spectrum of biological functions and applications due to their capacity to have noncovalent interactions with several enzymes and receptors in living organisms. Hydrocarbons from coumarin are used in the therapeutic field to treat a wide range of diseases. There are several significant examples of coumarin used as an anti‐viral, anti‐fungus, antioxidant, anticancer, anti‐diabetic, and anti‐coagulant in the past few years. Furthermore, coumarins are used as fluorescence sensors in biological systems (Pereira et al. 2018).

Derivatives of coumarins (sulfonamide and benzamides) are useful anticancer agents as well. These potent have demonstrated a remarkable ability to modulate possible anticancer effects based on various substitution sequences (Rawat and Reddy 2022). Cancer is a serious illness that results from the uncontrollable and exponential proliferation of cells, which can cause diseases and organ failure (Rommasi and Esfandiari 2021). One of the most dangerous illnesses that pose significant risks to life is cancer. Cancer death rates are soon going to exceed those from heart disease and stroke. The WHO's report indicates that cancer is currently the leading cause of death all over the world. According to “GLOBOCAN” 9.56 million cancer deaths and 18.08 million new cases of cancer will be expected, with low‐ or middle‐income countries contributing to 70% of these deaths (Al‐Warhi et al. 2020).

Renal cell carcinoma, prostate cancer, and leukemia are common conditions that are treated with coumarin. It can also be used to mitigate the negative effects of radiation therapy. Due to its potential for photochemotherapy and other cancer treatments, coumarin derivatives, both natural (7‐hydroxycoumarin, aesculetin (6, 7‐dihydroxycoumarin), psoralen, and imperatorin) and artificial (coumadine and warfarin), are popular (Akkol et al. 2020).

**FIGURE 1:**
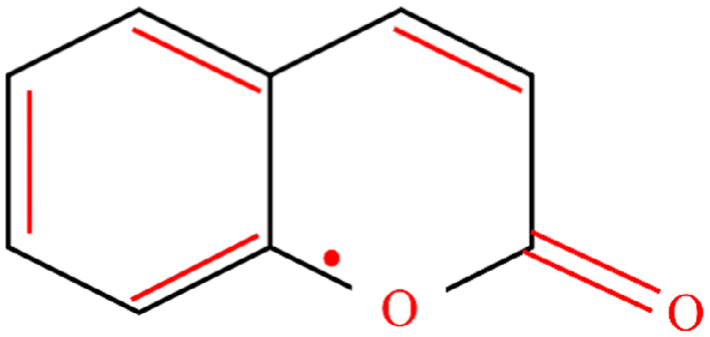
Chemical structure of coumarin.

A large number of coumarin derivatives (umbelliferone, esculetin, and quercitin) are accessible, and their pharmacological properties, commercial availability, and riches in medicinal plants fulfill significant roles as drugs in several medical domains (Sharifi‐Rad et al. 2021). Figure 1 shows the chemical structure of coumarin.

## 2 Antioxidant Status of Coumarin

Many coumarin compounds have been shown to have significant antioxidant qualities (Lončarić et al. 2020). Coumarins are present in daily consumed foods, including wine, tea, coffee, almonds, and olive oils. It can be used as spices, herbal teas, or medicine (Stassen et al. 2021).

Using diverse methods, including free radical scavenging, antioxidant enzyme activity enhancement (catalase and superoxide dismutase), and transcription factor modification related to oxidative stress, coumarins have substantial antioxidant effects (Katopodi et al. 2021). Reactive oxygen species (ROS) overproduction leads to oxidative stress, which is a major factor in the etiology of numerous diseases, including neurological and cardiovascular illness, tumor growth, and age‐related conditions (Alshibl et al. 2020). Free radicals are transient, highly liable molecular species distinguished by the presence of unpaired electrons. They can harm lipids, proteins, carbs, nucleic acids, and carbohydrates and cause many non‐communicable diseases. By lowering oxidative stress, antioxidants scavenge these free radicals and prevent cellular damage, which is advantageous for human health. Increased consumption of antioxidant‐rich foods as dietary supplements has already been shown in epidemiological studies to lower the risk of numerous illnesses. The study of natural antioxidants and their impact on nutrition and human health is an emerging subject (Chandra, Sharma, and Arora 2020). Anthocyanin (36% coumarin) from *Aronia melanocarpa* may be useful in preventing Alzheimer's disease, according to a study. When this was applied to SH‐SY5Y cells in an in vitro model of Alzheimer's disease, they prevented Aβ1‐42 induced apoptosis, reduced intracellular ROS and Ca^2+^, and raised ATP and mitochondrial membrane potential (Pîrjol et al. 2023). After 7‐hydroxy‐4‐methyl coumarin was alkylated in two steps, a novel coumarin derivative known as 7‐((8‐(4‐benzylpiperidin‐1‐yl) octyl) oxy)‐4‐methyl‐2H‐chromen‐2‐one (C3) was isolated. Using viscosimetric and UV–vis spectrophotometric techniques, the DNA binding relationship with C3 was assessed. The outcomes of these studies demonstrated that C3 was interactively bonded. Using the DPPH technique, C3's antioxidant activity was assessed. The analysis showed that C3 can eliminate DPPH radicals. Considering the enzymes EGFR and CYP450 as receptors, molecular docking was used to examine the potential mechanism of C3's antioxidant and anticancer action. Additionally, the C3 tended to have high antioxidant potential with good binding affinity values (−7.82 kcal/mol) and binding site interactions, according to the molecular docking research analysis. The chemical C3 demonstrated effectiveness as an antioxidant and anticancer therapeutic candidate based on all experimental and theoretical evidence (Kecel‐Gunduz et al. 2023).

Polyphenolic coumarins are known to have antioxidant properties in biological systems; it can be challenging to figure out this property from the variety of additional impacts they have on cells. Electron paramagnetic resonance spectroscopy was used to quantify the radical scavenging capabilities of 22 structurally similar natural and synthetic 4‐methyl coumarins by evaluating their reaction with radicals, galvinoxyl and 2, 2‐diphenyl‐1‐picrylhydrazyl. By utilizing the DCF fluorescent probe test to measure the quantities of reactive oxygen species inside cells, the effective antioxidant activity of 4‐methyl coumarin was confirmed. It was anticipated that o‐dihydroxysubstituted coumarins would be superior to the m‐dihydroxysubstituted or monohydroxysubstituted counterparts as superior radical scavengers. However, it was unexpected that even in the lack of esterase, the equivalent o‐diacetoxy derivatives would function effectively as scavengers (Pedersen et al. 2017). Fruits possess a multitude of salutary effects on human health and exhibit robust antioxidant activity. However, the bio‐accessibility and bioavailability of these advantageous bioactive chemicals (polyphenols, flavonoids, and carotenoids) determine how well the human body can use them. Natural secondary metabolites have the potential to reduce inflammation and act as antioxidants, which help to prevent diabetes, cancer, and heart diseases. The pathogenesis of several clinical illnesses is largely dependent on the unregulated essential cellular pathways caused by oxidative stress, which is stabilized by natural antioxidants. The majority of the publications examined the novel role that naturally occurring antioxidants play in preventing disease. Since adopting a healthier lifestyle has become popular, researchers are looking for more naturally occurring antioxidants with powerful therapeutic potential to eventually replace some manufactured medications that have undesirable side effects It concluded that the synthesis of natural antioxidants is crucial for both preventing and treating human diseases as well as for reducing any adversity that compromises the health (Ramana et al. 2018).

## 3 Pharmacokinetics Study of Coumarin

The study of pharmacokinetics involves the biological processes of absorption, distribution, metabolism, and excretion, and it focuses on the dynamic movements of foreign chemicals, or coumarins, as they transit through the body. Numerous pharmacological characteristics of natural coumarins, such as their anti‐inflammatory, antioxidant, neuroprotective, and anti‐tumor activities, have been studied (Srikrishna, Godugu, and Dubey 2018). After consuming coumarins orally, they are rapidly absorbed from the gastrointestinal tract (GIT) and dispersed throughout the body. Both 7‐hydroxycoumarin and coumarin are weakly dissolved in water. Due to their non‐polar nature, it was identified that coumarins can readily diffuse through lipid double layers. According to a clinical pharmacokinetic study, 7‐hydroxycoumarin was fully absorbed from the gastrointestinal tract and rapidly processed by the liver in the first pass; only 2%–6% of it made it into the systemic circulation. Coumarin, the precursor of 7‐hydroxycoumarin, has a short half‐life and limited bioavailability. Pharmacokinetic analyses revealed that 47% of 7‐hydroxycoumarin and 35% bind to plasma proteins (Akkol et al. 2020).

The human liver undergoes substantial metabolism of the dietary component coumarin to produce excretable 7‐hydroxycoumarin. Despite being considered safe when consumed in food on a daily basis. Coumarin has both toxicological and clinical significance due to its possible link to hepatotoxicity, which is particularly noticeable in rats. The human virtual oral administration of coumarin served as a framework for the pharmacokinetics of the drug. The biotransformation of coumarin to o‐hydroxyphenylacetic acid (via 3, 4‐epoxidation) and the modified monitoring equivalents of coumarin were determined using specified species allometric scaling constants. Human coumarin equivalents were calculated using the available plasma concentrations from rat research. Rat and human liver preparations were used to gather data on the rapid in vitro metabolic clearance for humans (about 50 times faster than in rats) for in vitro and in vivo interpretation. To replicate human physiologically based pharmacokinetics (PBPK), the metabolic ratios to 7‐hydroxycoumarin and o‐hydroxyphenylacetic acid were set at major (0.9) and minor (0.1) levels, respectively, to account for the complete elimination of coumarin. The human plasma concentration curves that were produced by using PBPK models were by the maximal predicted concentrations of coumarin that have been reported. Since there was minimal evidence of coumarin's toxicological effects in people under the prevailing assumptions, advanced dosage utilizing PBPK modeling proved helpful in assessing human risk (Miura et al. 2020).

Esculetin is a dihydroxy coumarin found naturally. Numerous in vivo and in vitro research on esculetin has revealed significant pharmacological activity. Esculetin primarily functions as an antioxidant, anti‐apoptotic pharmacological agent. Additionally, esculetin's wide range of biological actions makes it a viable pharmacological prospect for the treatment of several indications related to certain diseases, such as cancer, diabetes, and its consequences atherosclerosis, and nonalcoholic fatty liver disease among others (Zhang, Tan, et al., 2021; Zhang, Xie, et al., 2021). The researchers conducted a pharmacokinetic study in which the comparison between the oral and intravenous administration of coumarin solution in humans with prolonged‐release tablets containing coumarin was conducted. The relation between the area under the curve (AUC) and the percentage of drugs released in vitro was found. According to a study, a coumarin‐based prodrug system increased the oral bioavailability of meptazinol in rats by a factor of four. A different kinetic investigation revealed that the absorption of coumarin from powdered cinnamon is somewhat less than that of coumarin that has been separated (Sharifi‐Rad et al. 2021).

## 4 Anticancer Perspectives

A wide range of cancers, such as leukemia, malignant melanoma, prostate, breast, lung, and colon cancers, can be effectively treated by the anticancer characteristics of coumarin and its derivatives (Bhattarai et al. 2021). Coumarins are made of fused benzene ring and pyrone ring systems. Depending on the alterations made to the fundamental nucleus, coumarin has great anticancer potential with little side effects. When it comes to specific anticancer activity, coumarins have a remarkable capacity to modulate a variety of cellular pathways (Thakur, Singla, and Jaitak 2015). Research on coumarins and their derivatives' anticancer properties has shown that caspase‐dependent apoptosis is often these drugs' mode of action. By decreasing Bcl expression in a variety of organs and tissues, 7‐hydroxycoumarin, which is produced when coumarin is metabolized by CYP 2A6, an isoform of cytochrome P450, has an anti‐proliferative impact. The 26 kDa membrane protein Bcl inhibits CYP in the mitochondria, reduces the activation of caspase‐9, and blocks free oxygen radicals, all of which cumulatively lengthen the cell life cycle. Consequently, it induces carcinogenesis and causes oncogenic mutations to accumulate within healthy cells. Protein Bax is a membrane protein that activates caspase‐9. By altering the mitochondrial membrane, overexpression of Bax releases the mitochondrial cytochrome C into the cytoplasm. Important nuclear cytoplasmic proteins are broken down by caspase‐9, which is activated by cytochrome C in the cytoplasm. This process also activates caspase‐3, 6, and 7 (Akkol et al. 2020).

**FIGURE 2:**
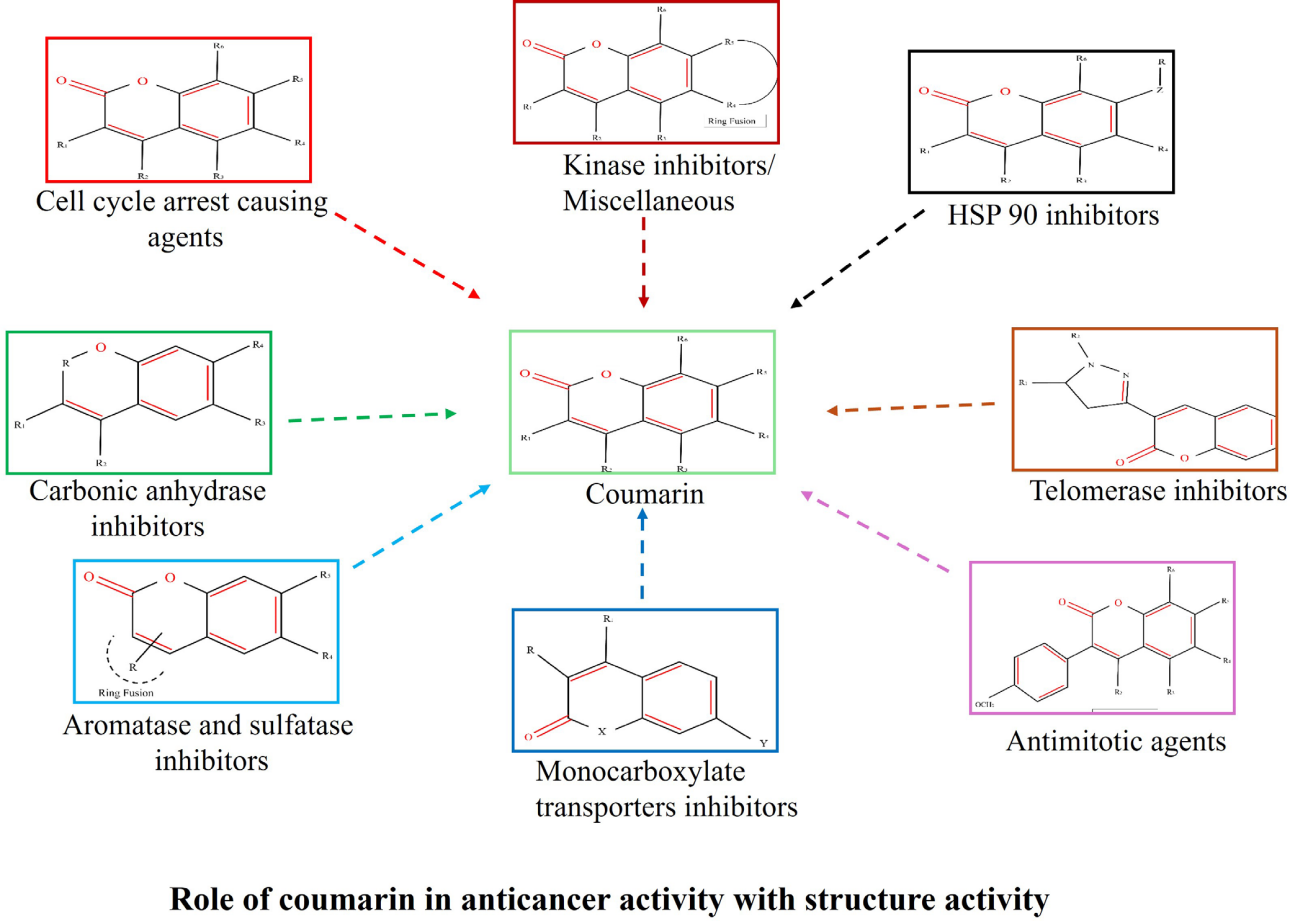
Role of coumarin in anticancer activity with structure–activity.

Many classes of coumarin‐based anticancer agents, including alkylating agents, kinase inhibitors, angiogenesis inhibitors, telomerase inhibitors, topoisomerase inhibitors, antimitotic activity, human carbonic anhydrase inhibitors, aromatase inhibitors, mono‐carboxylate transporter inhibitors, and hormonal antagonists, have been thoroughly studied by the researcher. Both in vitro and in vivo, coumarin compounds from a variety of natural sources show encouraging anticancer efficacy. Therefore, combining coumarin and artemisinin is a good way to synthesize anticancer medications that work better (Koley et al. 2024). Figure 2 illustrates the role of coumarin in anticancer activity with structure–activity.

In order to formulate potent anticancer medications, researchers produced six different kinds of coumarin derivatives in 2011. These included quaternary ammonium coumarins, pyrano‐coumarins, coumarin carboxamides, 3‐alkyl‐4‐methylcoumarins, 7‐aminocoumarins, and 4‐aminocoumarins. In addition to studying the chemical's ability to inhibit kinase, the researcher also looked at their ability to limit cell proliferation. The research team proposed that the anti‐proliferative activity and the kinase inhibitory potency have a weak connection (Thakur, Singla, and Jaitak 2015). Because azole has a five‐membered ring with one nitrogen atom and at least one other non‐carbon element (such as oxygen, sulfur, or nitrogen), it is regarded as one of the most significant heterocyclic compounds. The coumarin molecule has been associated with a number of azole types to produce new anticancer medications with improved anticancer properties because of their significant biological effects (Zhang 2020). Coumarin is one of the most extensively used bioactive components for the development of anticancer drugs. It inhibits tumor growth through a variety of methods and has adaptable anticancer properties. Target‐based coumarin derivatives are also preferred to be made using simple synthetic approaches. By using the coumarin substitution pattern, “structural Activity Relationship (SAR)” experiments identify anticancer potentials with few side effects. Many compounds containing coumarin have been synthesized for their use in cancer therapy against key targets such as tubulin protein, carbonic anhydrases, and galectin (Gal‐1) (Goud, Kumar, and Bharath 2020). Due to inadequate diagnosis, a lack of effective choice treatments, and limited prevention measures, the rate of cancer mortality is alarming. Despite its extensive distribution in numerous naturally occurring substances, coumarin‐privileged scaffolds possess an exceptional anticancer profile. This led to a thorough investigation of the anticancer potential of several families of coumarin derivatives using the mode of action. To enhance the quality of life, particularly for cancer patients, novel compounds that have been extracted from natural sources including plants and animals, as well as the potential combining of these compounds with traditional chemotherapeutic treatments, appear to be crucial (Rawat and Reddy 2022).

## 5 Pancreatic Cancer

All over the world, the sixth most common cause of death is pancreatic cancer. According to the Global Cancer Observatory estimation, there were 495,773 new cases and 466,003 deaths occurred globally in 2020. Pancreatic cancer mortality and incidence are rising over the past few decades. About 6% of people survive for 5 years, with industrialized and developing nations having slightly different rates (Mizrahi et al. 2020). Each year, almost 300,000 patients lose their lives to pancreatic cancer. Pancreatic ductal adenocarcinoma is the most aggressive and lethal type of pancreatic cancer, and < 5% of patients live for 5 years (Lu et al. 2017). Combining two or more non‐identical pharmacophores into a single molecule is known as molecular hybridization. It has become a viable approach that enables the creation of molecular frameworks that are more active and have a higher affinity than their parent medication. A copper‐catalyzed azide‐alkyne cycloaddition (CuAAC) was used in the study to synthesize and analyze two new hybrids that unite antioxidant coumarin derivatives (4‐azido coumarin and coumarin‐1,2,3‐triazole hybrids) with well‐known anticancer chemotherapeutic drug with 5‐fluorouracil. The conjugates demonstrated strong antioxidant capabilities as well as an ability to combine and create stable, uniformly sized, regular non‐particles in aqueous conditions. When tested in vitro against PANC‐1 human pancreatic cancer cells, these substances showed exclusive toxicity and outperformed free 5‐fluorouracil in terms of activity. It was concluded that 5‐fluorouracil and coumarin derivatives (4‐azidocoumarin) work collaboratively, and they also call for more research into these hybrids as potential pancreatic anticancer treatments (López et al. 2022).

It was previously found that novel isoprenylated coumarin compounds exhibit specific cytotoxicity against the pancreatic cell line PANC‐1 only when glucose is low. Using the cell cytotoxicity assay, the researchers examined the anti‐proliferative mechanism of the most powerful isoprenylated coumarin molecule in the series, DCM‐MJ‐I‐21, against two more pancreatic cancer cell lines, BxPC‐3 and CAPAN‐2. The amount of autophagic flux impacted by drugs, autophagy inducers, and autophagy inhibitors was measured by using western blotting. Since glycolysis is not a viable survival strategy for BxP3 and CAPAN‐2 pancreatic cell lines, a definite requirement of glucose was found in DCM‐MJ‐I‐21. It indicates that a chemical targets the route shared by these cancer cell lines. In PANC‐1, the lead compound in the drug enhanced the conversion of LC3‐1 to LC3‐2, a function analogous to that of autophagy inhibitor chloroquine. Furthermore, in both 2D and 3D cell cultures, another autophagy inhibitor called Spautin‐1 had nearly the same anti‐proliferative action at the same concentration when subjected to the nutrient‐deprived circumstances as the lead drug. It was concluded that by inhibiting autophagy, the lead isoprenylated coumarin molecule selectively kills pancreatic cancer cells in nutrient‐starved environments, thereby offering fresh therapy possibilities (Zhou, Kusaka, et al. 2022; Zhou, Yu, et al. 2022).

The NEDD8‐activating enzyme (NAE) activates the ubiquitin‐like protein Neural precursor cell Expressed Developmentally Downregulated protein 8 (NEDD‐8). Many cancers and inflammatory illnesses can be caused by the overexpression of NAE. To achieve therapeutic objectives, the rate of ubiquitination and the consequent degradation of cancer‐associated proteins could be mediated by specific suppression of NAE. In the research, the researcher chose to investigate the production and screening of coumarin derivatives and scaffolds against cancer cell lines, namely the human BxPC‐3 human pancreatic cancer line. To weed out potential compounds, 24 targeted compounds were produced and tested for cytotoxicity against three normal cell lines using the MTT tests and anti‐proliferative activities against three cancer cell lines. Subsequently, research on cell death and enzyme and cell‐based assays verified the target even further. The majority of the newly synthesized 4‐position modified coumarin shows anti‐proliferative activity against the three cancer cell lines. Several tests were run to determine which compound coumarin scaffold was the best option. With an IC‐50 value of 0.28 micro‐moles, this molecule demonstrated the maximum effectiveness against BxPC‐3 cells.

**FIGURE 3:**
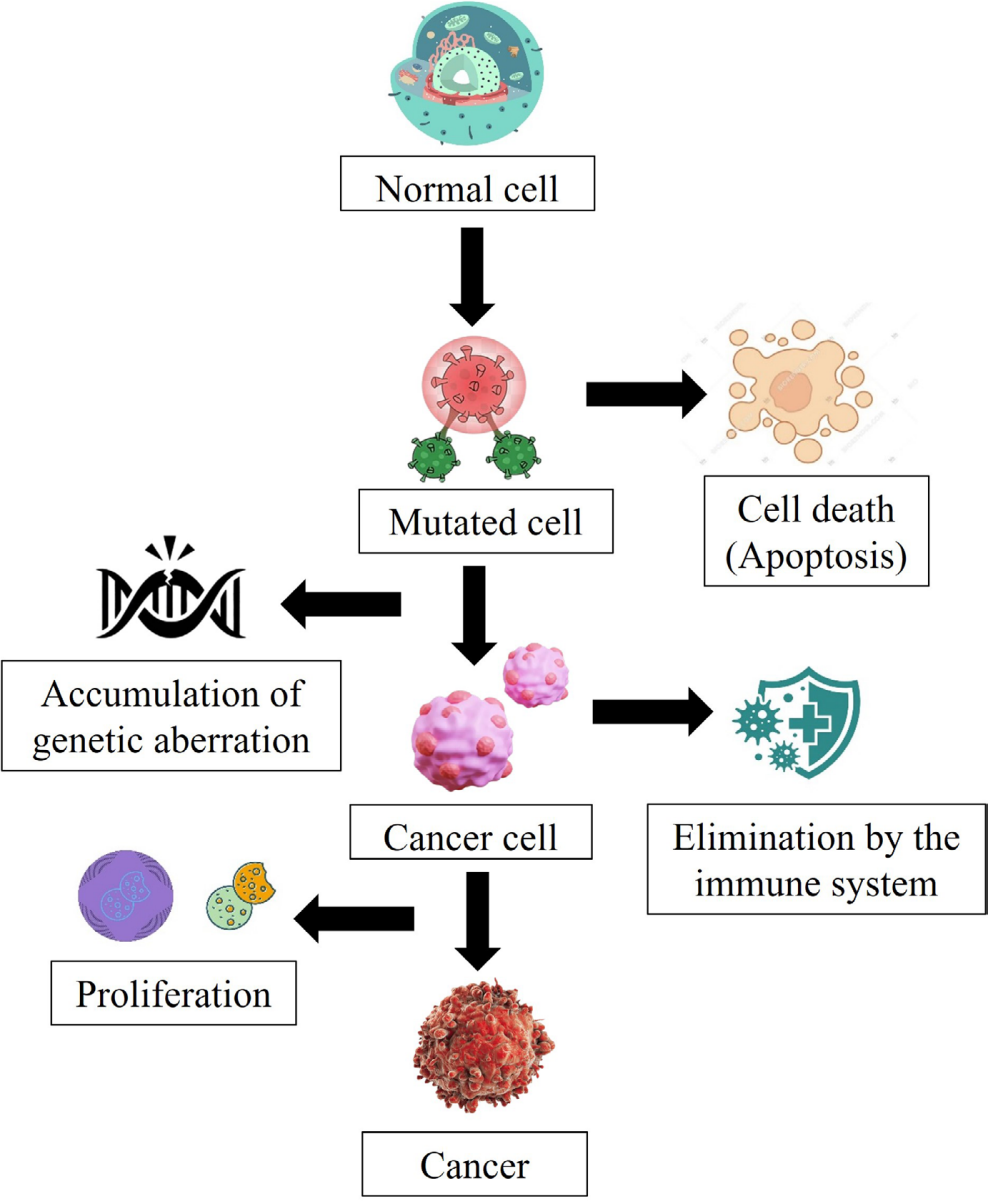
Mechanism of carcinogenesis.

Additionally, it causes the BxPC‐3 cells to undergo apoptosis and accumulate the substrate through the action of CRLs, as well as limit the NAE function in enzyme and cell‐based assays. In three normal cells, however, its toxicity was rather minimal. It was concluded that the coumarin compound caused apoptosis in BxPC‐3 cells and hindered NAE activity in enzyme and cell‐based systems. Furthermore, its toxicity was modest. These findings implied that the coumarin scaffold would be a promising starting chemical for the creation of anticancer medication (Gong et al. 2022). The mechanism of carcinogenesis is presented in Figure 3.

## 6 Colon Cancer

Colon cancer is a leading cause of morbidity and death worldwide. It is the second most frequent reason for death from cancer. In 2018, there were 881,000 deaths worldwide and almost 1.8 million new cases. Due to the improvement in early identification and treatment, the death rate from colon cancer has decreased recently; although five‐year survival rates at advanced or metastatic stages are still poor. When the disease is in its early stages, the 5‐year survival rate is around 90%; however, as disease progresses, the survival rate drops to about 10% (Siegel, Miller, and Jemal 2018). Consequently, there is an urgent need to comprehend the molecular mechanism behind the development of cancer cells (Favoriti et al. 2016). According to the report by the “American Cancer Society,” colon cancer is one of the lethal tumors and is thought to have killed over 50,000 people in the United States in 2018. One of the coumarin compounds with methoxy on C7, osthol has been shown to have potent anti‐tumor properties. Osthol appeared to have a tissue‐specific effect on colon cancer cells (HCT116 and SW480cells) among a variety of tumor types, with a dose of osthol applied that was significantly lower than that of other cancer cells such as prostate and breast cancer cells (Lin et al. 2019).

Using a bioisosteric transformation technique, researchers developed new geiparvarin anticancer analogs. Additionally, the investigation demonstrated that the benzene rings’ cytotoxic effects were enhanced when electron‐withdrawing substituents such as 7‐((1‐(‐4‐flourobenzyl)‐1‐H‐1, 2, 3‐triazole‐4‐yl) methoxyl)‐4H‐chromen‐4‐one were added. This was in comparison to geiparvarin, which had an IC50 in the cases of human hepatoma and colon carcinoma cell line (Akkol et al. 2020). Dicoumarol (DIC), a coumarin derivative with some anticancer properties that is involved in redox modulation, was also examined. It was discovered that when colon cancer cells overexpressed HMGA2, DIC might cause apoptosis and prevent the cells from migration. In colony‐formation tests, DIC may also enhance 5‐FU's anti‐tumor effects. It offered a fresh perspective on the molecular role of HMGA‐2 and proposed a potential therapeutic use of DIC to stop the spread of colon cancer cells that overexpress HMGA‐2 (Chen et al. 2020).

Genetic alterations present in the tumor have an impact on cancer treatment, and the prognosis is still bad at an advanced or metastatic stage. For example: the KRAS gene mutation presents a therapeutic difficulty for EGFR‐targeted therapies; so, other strategies are required to lower the failure rate in colon cancer therapy. It may be possible to use coumarin or its synthetic or biological variants. Researchers have produced five derivatives of coumarin. These derivatives were triflourimethyl, dimethoxy, and nitro‐substituted. It was revealed that “5, 7 dimethoxy‐4‐methyl‐6‐nitro‐chromen‐2‐one,” a nitro coumarin derivative, exhibited the maximum cytotoxicity and incited apoptosis in cancer cells. It can also reduce colon cell migration and prevent both long‐term and short‐term proliferation. When applied to colon cancer cells that expressed either wild‐type or mutant KRAS genes, it showed effectiveness (Lu et al. 2020).

Osthol and imperative are two examples of active coumarins found in a variety of plants. The study examined the cytotoxic effect and mode of action of two naturally occurring coumarins, imperatorin and osthol, when administered to human colon cancer cells both alone and in conjunction with 5‐fluorouracil (FU). In order to screen for appropriate concentrations of osthol, imperatorin, 5‐FU, and API‐1 with cytotoxic and cytostatic potential and to constantly track cell survival and proliferation in CoLo‐205 cells, the xCELLigence System was used for real‐time cell analysis. Drug effects on p38MAPK‐alpha levels and mRNA expression were also evaluated. Osthol increased 5‐Fu's cytotoxic effects and continuously showed potent anti‐proliferative action against CoLo cells. Osthol was identified as the protein kinase inhibitor API‐1. Osthol, osthol + 5‐FU, and imperatorin treatments significantly raised the phosphor‐p38MAPK‐alpha/p38MAPK‐alpha ratio. It was demonstrated that ostho l,5‐FU and API‐1 + 5‐FU significantly decreased the expression of Akt mRNA in comparison to the positive control API‐1. According to the results, osthol shows interest as an anticancer agent for colon cancer treatment. It may also boost the effectiveness of 5‐FU which could lessen the frequent adverse effects of standard chemotherapy when used in conjunction with 5‐FU for colon cancer treatment (Karaboğa Arslan et al. 2021).

## 7 Prostate Cancer

Among men, prostate cancer is the second leading cause of cancer‐related mortality. In 2020, there were 1,414,259 new cases and 375,304 deaths reported worldwide. After receiving a variety of treatments, including radiation, hormone therapy, prostatectomy, and even active surveillance, many individuals with early‐stage cancer have a prognosis; nevertheless, the prognosis for patients with late‐stage and metastatic prostate is still difficult to predict (Elagawany et al. 2024). New therapeutic approaches are needed because prostate cancer is one of the main causes of death for men. The researchers evaluated esculatin, a coumarin derivative, for its anti‐migration and anti‐proliferative properties. The human prostate cancer cell line's vitality was evaluated using the MTT test after being treated with different dosages of esculatin for 24–72 h. The cell cycle and apoptosis were investigated using a cell‐based cytometer. Gene expression levels were assessed using quantitative real‐time PCR and reverse transcription, while cell mobility was assessed using the wound healing assay. Protein expression was measured by western blotting. Esculetin inhibited cell proliferation in a dose‐ and time‐dependent manner. Treatment with esculetin reduced cell migration. The administration of esculetin resulted in a phase cell cycle arrest, apoptosis, and a considerable reduction in cell survival as demonstrated by an image‐based cytometer. The analysis indicated that PCa (prostate cancer) may be managed with the coumarin derivative esculetin. Still, more in vivo studies or research are required (Turkekul et al. 2018).

Coumarin and benzimidazole have distinct biological properties such as anticancer properties. Coumarin‐benzimidazole hybrids were produced and their potential anticancer effects were explored. 4‐Chloromethylene coumarin and N‐benzyl benzimidazole derivatives reacted to form a 16‐coumarin substituted benzimidazolium chloride. These compounds were all analyzed using elemental analysis, IR spectroscopy, and NMR. All compound's toxicities were assessed using MTT (2, 5‐diphenyl‐2H‐tetrazolium bromide) assaying prostate cancer cells. At 100 μm, every chemical showed notable cytotoxicity against cancer cell lines. Furthermore, certain compounds exhibited noteworthy actions at 1 μm against prostate cancer cell lines, and acquired outcomes indicated that these kinds of drugs are an attractive option for the treatment of prostate cancer (Karataş et al. 2019). In another research, to separate the bioactive component sesquiterpene coumarins from an asafetida dichloromethane extract, several chromatography studies were performed. Through analysis of the 1H‐NMR spectra and comparison with structures reported in the literature, the structures of the isolated compounds were made clear. Resazurin and a non‐fluorescent substrate were used in the experiment to evaluate the cytotoxic activity of pure compounds. The cytotoxic effect of pure compounds was measured using the non‐fluorescent substrate resazurin. Different amounts of pure chemicals were applied to human prostate cancer cells. For the purposes of this study, 10 sesquiterpenes were isolated from the oleo‐gum‐resin of *Ferula assa‐foetida* , and the cytotoxic potential of six of the compounds was assessed using MCF‐7, PC‐3, and NIH cell lines. Gummosin, a sesquiterpene coumarin, showed mild cytotoxic activity against PC‐3 and MCF‐7 cell lines, with IC50 values of 30 and 32.1 μg/mL, respectively. Cancer cells did not exhibit any toxicity toward any isolated compounds (Iranshahy et al. 2019).

A cyclometalated IrIII complex conjugated to a coumarin that produces far‐red light, known as IrIII‐COUPY, has recently been shown to be a high‐potential photosensitizer suitable for photodynamic therapy of cancer. The primary goal of this work was to better understand the mechanism underlying a new class of drugs that may be developed as therapeutic possibilities for incredibly rare human malignancies, such as androgen‐resistant prostatic tumors of metastatic origin. These compounds have a photoactive anticancer effect. IrIII‐COUPY is a far‐red light‐emitting cyclometalated IrIII complex attached to a coumarin. It is a highly promising photosensitizer that can be applied to cancer photodynamic therapy. Simultaneously and comparably, it effectively eradicates prostate mass, differentiated, prostate, and almost incurable stem cells. Consequently, photodynamic therapy and other photo‐induced therapies using IrIII‐COUPY conjugates may offer an entirely new strategy for treating prostate cancer (Novohradsky et al. 2021).

## 8 Breast Cancer

Among the malignancies that affect women most frequently worldwide is breast cancer. With an estimated 12% lifetime risk, breast cancer is the second leading cause of mortality for women. Mammary cell proliferation and positivity for the progesterone, estrogen, and human epidermal growth factor receptor 2 receptors have historically been used to classify breast cancer. Clinical research has demonstrated the great efficacy of ER antagonist‐therapeutic drugs that block the function of estrogen in the treatment of breast cancer. A class of phytochemicals known as coumarins has a variety of pharmacological properties and is used in photo‐chemotherapy, anticancer, and anti‐HIV therapies, among other therapeutic applications. According to certain studies, coumarins have a little estrogenic effect in delaying the onset of menopausal‐related illnesses (Dhawan et al. 2018).

Many coumarins and their active metabolite 7‐hydroxycoumarin analogs have demonstrated potential in the treatment of breast cancer due to their capacity to inhibit sulfatase and aromatase. Furthermore, it has been suggested that coumarin estrogen conjugates and coumarin‐based selective estrogen receptor modulators (SERMS) may serve as preventative strategies against breast cancer. Potential new medication treatments are desperately needed, as breast cancer is the second greatest cause of death for American women, after lung cancer (Musa, Cooperwood, and Khan 2018). Coumarin is found in many medicinal plants, and its therapeutic qualities have been identified. It was identified that coumarin exhibits a variety of biological actions and has beneficial effects on human health. 1‐N‐(acetyl)‐azacoumarin‐3‐carboxylic acid and 1‐N‐(2‐formyl‐1‐phenyl) vinyl were the synthetic forms of substituted azacoumarin‐3‐carboxylic acids used in the study. These compounds include 1‐N‐(4‐cyano‐5‐methoxy‐5‐oxo‐1‐phenylpenta‐1,3‐diene‐1‐y), azacoumarin‐3‐carboxylic acid, azacoumarin‐3‐hydroxycysteine, and 1‐N‐[2‐(hydroxy) caybonyl‐1‐(phenyl) vinyl]. The chemical structures of these compounds were investigated by means of proton NMR spectroscopy, infrared, and elemental analysis. The cytotoxic potential of all the synthesized derivatives against the MCF‐7 cell line was examined (Gaber et al. 2021). KB (human oral epidermoid carcinoma cell line), HCT‐116 (human colon cancer cells), and MDA‐MB‐231 (human breast cancer cell line) were three human cancer cell lines used in the MTT assay to assess the in vitro toxicity of these synthesized compounds. 5‐A positive control was added in the form of fluorouracil in coumarin derivatives. The investigation showed that most of the compounds have a strong inhibitory effect against MDA‐MB231 (breast cell line) and KB‐cell lines. In particular, coumarin sulfonamide and coumarin amide demonstrated a strong inhibitory effect against breast cancer cell lines. It was also noted that 7‐diethylaminocoumarin sulfonamides showed more activity than 7‐alkoxycoumarin sulfonamides among coumarin sulfonamides, suggesting that the diethyl‐amino group at the C‐7 position increased potency. When compared to the amide derivatives, coumarin sulfonamide expressed superior cytotoxic activity against cancer cells.

Moreover, it was also demonstrated that with the longer treatment times, the scratch gaps in MDA‐MB231 cells treated with compound coumarin sulfonamide were wider than those in the control group. In comparison to the control group, the wound area increased and the inhibition of cell migration occurred in a concentration‐dependent way. This study concluded that altering the structure of coumarin sulfonamide gave the most anti‐proliferative effects against MDA‐MB231 cells. One of the hallmarks of tumor metastasis is the ability of tumor cells to move and invade adjacent tissue. The effects of compound coumarin sulfonamide on the migration and invasion of MDA‐MB231 cells have been further investigated. Compound coumarin sulfonamide treatment significantly decreased the proportion of MDA‐MB231 cell migration and invasion (Zhang, Tan, et al., 2021; Zhang, Xie, et al., 2021).

The project's design, growth, and development, as well as the in vitro testing of numerous novel pharmacophores comprising fatty acids and coumarin compounds for the prevention of breast cancer, were provided by the researcher. They used a structure‐based approach to drug design and generated a library of coumarin‐fatty acid conjugates. The conjugates exhibiting encouraging in silico outcomes were subsequently synthesized, characterized, and evaluated via the utilization of MTT, apoptotic, cell death, cell growth, estrogen binding, and gene expression tests to ascertain their effectiveness against breast cancer. SAC‐2 and LNAC‐2, two of the fifteen compounds evaluated, showed good activity, with IC‐50 values of 22 and 25 μg/mL, respectively. These compounds accelerated the breakdown of ER‐alpha, deactivating the ER‐alpha pathway and preventing the proliferation of MCF‐cells overexpressing ER. The expression of the ER‐alpha receptor and the AKT‐1 gene was inhibited by SAC‐2, according to the outcomes of RT‐PCR and ER‐binding assays on gene expression. Because compound SAC‐2 is an effective ER antagonist, it may be used to treat breast tumors and other cancers where AKT plays a substantial role (Selvaraj et al. 2020).

## 9 Kidney Cancer

Renal cell carcinoma or RCC is the most common kidney cancer originating from the renal tubules and makes up around 85% of kidney cancers that are malignant. Approximately, 14,000 people lose their lives to RCC each year and more than 60,000 new cases are reported (Haque et al. 2017). Despite all of their uses in the search for bioactive substances, coumarin continues to be one of the most adaptable classes of substances for the development of anticancer drugs. Numerous researchers have looked into the potential application of coumarin in the therapy of cancerous cells. The in vitro investigation of coumarins against renal cell cancer demonstrated the cytotoxic and cytostatic properties of coumarin and 7‐hydroxycoumarin (Emami and Dadashpour 2015).

The researcher conducted dosage and toxicity tests which showed that coumarin was well tolerated at the applied dose (500–600 mg). The strong scent of coumarin was assumed to be the cause of nausea which was identified as a common adverse effect (Akkol et al. 2020). The current investigation shows the anticancer properties of an original drug indolyl‐coumarin called COUFIN, with a focus on how effective it is against clear cell carcinoma. COUFIN was attached to the tubulin at or near the colchicine site and hindered the production of microtubules. Renal carcinoma in both monolayer and multilayered tumor spheroid (3D culture) cultures shows anticancer activity against COUFIN. It was investigated that indolyl‐coumarin acts as a bifunctional molecule because without effluxes by ATP‐binding Cassette proteins (ABC) protein, it can act as an anti‐proliferative agent. COUFIN thus appears to be a potentially effective chemotherapeutic drug for tumor cells that overexpress efflux pumps as well as tumor cells irrigated by endothelial‐lined arteries which are accountable for the poor dispersion of traditional anticancer medications (Champelovier et al. 2014).

Combining the coumarin component with other anticancer pharmacophores is a privileged approach to enable beneficial therapeutic approach for cancer therapy. The liver and kidney's overall antioxidant capacity was assessed. Additionally, the tissues of the liver and kidney were examined histo‐pathologically. The injection of the test substance neutralized the majority of the degenerative changes caused by “Ehrlich ascites carcinoma cells (EAC)” cells in mice, according to the results of tests on kidney and liver function as well as histological analysis. These results allow for the development of the test chemical as a potent chemotherapeutic agent (Mohammed et al. 2020).

## 10 Bladder Cancer

Bladder cancer is the ninth most common type of cancer worldwide. The significant burden on health services is caused by its high frequency, susceptibility to numerous recurrences, and progression even with local therapy. African countries with higher rates of schistosomiasis infections are more likely to have bladder cancer with squamous cells. The most common histologic kind of bladder cancer is urothelial carcinoma. The main causes of bladder cancer are smoking tobacco, being exposed at work to chemicals that might cause cancer, like carbon black dust and aromatic amines, and drinking water that has been chlorinated or contaminated (Wong et al. 2018).

Many forms of cancer have been reported to be treated by coumarin and its derivatives, significant phytochemicals found primarily in higher plants (Önder 2020). Despite the possible medicinal uses of these substances, some 4‐methyl coumarins were created to study their anti‐tumor cytotoxic effects on the human bladder cancer cell lines T24 and RT4. With the changes at position 7, the coumarins were synthesized using microwave assistance and produced high yields using “Pechmann condensation.” It was hypothesized that the 5‐carboxy coumarin ring group positively affects the cytotoxic activity based on the chemical structures of the substances. It concluded that further chemical modification of these novel compounds may yield a new anticancer drug (Rocchetti et al. 2022).

## 11 Liver Cancer

The sixth most frequent cancer worldwide and the fourth most common in Egypt is hepatocellular carcinoma. Among the most malignant solid tumors, HCC is the third largest cause of cancer‐related death globally. Patients with HCC have a dreadful prognosis: approximately 80% of them die within the year of receiving their first diagnosis and just 18% of them survive for 5 years (Issa et al. 2022).

The disadvantages of anticancer medications used to treat liver cancer include their effectiveness in the disease's later stage, their toxicity to healthy cells, and the possibility of drug resistance. Several analogs of coumarin‐based drugs are presently being studied in preclinical and clinical trials, providing strong evidence that they are more effective than other anticancer medicines. About liver cancer, the current study sought to investigate the anti‐tumor effectiveness of a family of 8‐methoxy coumarin‐3‐carboxamides. Synthesized and characterized a novel set of N‐8‐methoxycoumarin‐3‐carboxamide analogs to achieve the goal, were created. Comparing the synthesized family of compounds to staurosporine, the evaluation of their anticancer activity showed that they were significantly more cytotoxic to Hep‐G2 cells while having no noticeable impact on normal cells. 8‐mthoxy‐azocoumarin‐3‐carboxamide, which was produced, exhibited the strongest cytotoxic effect against Hep‐G2 cells, with an IC‐50, surpassing the potency of the medication staurosporine. The mechanism of this compound is that it has an anti‐proliferative effect and is caused by DNA damage and interference with replication, which results in cell cycle arrest. This compound also caused necrosis and apoptosis in HepG‐2 cells, which is another way that it might cause programmed cell death, according to the research. Consequently, the recently synthesized family of chemicals, especially 8‐mthoxy‐azocoumarin‐3‐carboxamide, may offer a promising platform for the synthesis of anti‐tumor drugs to treat liver cancer (Radwan et al. 2023). The researcher used 75% ethanol to extract several coumarin compounds from *Juglans mandshurica* and then assessed the compound's toxicity in vitro using two different types of hepatocellular cancer cell lines. Several drugs exhibit mild anti‐tumor activity against these cell lines (Yao et al. 2017).

The diamine‐3 coumarin hybrid molecule, a coumarin‐based copper chelator, and its copper‐dependent macromolecule damage response were investigated by the researcher in lymphocytes overloaded with copper. The researcher used a rat model of diethyl nitrosamine‐induced hepatocellular carcinoma to examine the anticancer efficacy and mechanism of action of ligand‐L. It has been discovered that DEN‐induced hepatocellular carcinoma has a notable elevation in copper levels in liver tissues. Ex vivo results demonstrated that in isolated hepatocellular carcinoma cells, ligand‐L suppressed cell viability, generated reactive oxygen species, damaged DNA, lost mitochondrial membrane potential, and activated caspase‐3. Neocuproine and N‐acetylcysteine reversed every one of the effects that ligand‐L had caused. Furthermore, ligand‐L therapy of mice with hepatocellular cancer increases malignant hepatocytes' cellular redox consumers, lipid peroxidation, and DNA breakage. Overall, it indicated that the interaction between copper and ligand‐L generates ROS which in turn causes damage and malignant cell death. This offers sufficient evidence to establish ligand‐L as a lead chemical with clinical relevance for the treatment of various cancers (Khan, Zafar, and Naseem 2020).

**FIGURE 4:**
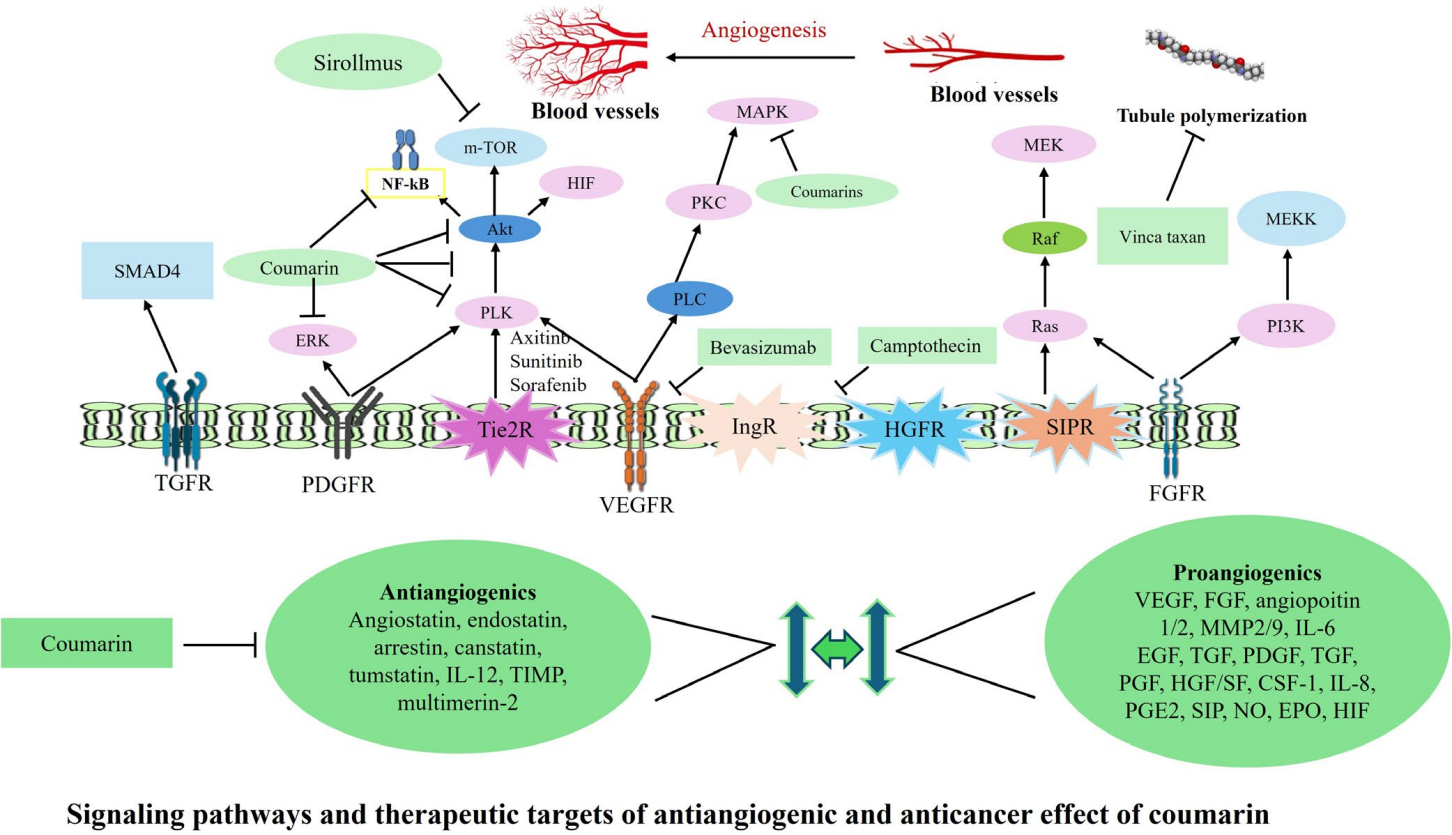
Signaling pathways and therapeutic targets of antiangiogenic and anticancer effect of coumarin.

Numerous poli‐clinic and clinical studies have shown that angiogenesis plays a crucial role in the initiation and spread of cancer. Coumarins are relatively non‐cytotoxic natural substances that have stronger antiangiogenic properties than traditional cytotoxic medication. Many intriguing antiangiogenic and non‐cytotoxic compounds were found by the utilization of semi‐synthesized and synthesized goods derived from organic coumarins as precursor chemicals. Coumarin possessing antiangiogenic and non‐cytotoxic characteristics nearly resembles the action of the psychological ligands associated with the primary therapeutic targets. It has been observed that many coumarin sulfonyl derivatives are cytotoxic and antiangiogenic to HepG2 hepatocellular carcinoma cells in vitro (Majnooni et al. 2019) (Figure 4).

## 12 Lung Cancer

Cells that divide uncontrollably, have heterogeneous genomes, and invade other tissues through lymph nodes or blood vessels are characteristics of cancer. Nearly 9 million cancer‐related fatalities occur each year, according to the reports of WHO. According to the “GLOBOCAN” data, lung cancer is one of the most frequent forms and has a death rate of 18.4% (Bray et al. 2018). Throughout the world, lung cancer is the leading cause of cancer‐related mortality and one of the malignant tumors with the highest diagnosis rate. Chemotherapy and radiation therapy are the main treatments currently available for advanced drug lung cancers although they are still insufficient. Although there are currently FDA‐approved medications that are very successful for therapy and chemotherapy, these approved medications have adverse effects (Konkoľová et al. 2021). The chemical combination of benzene and alpha‐prion ring characterized the class of secondary metabolites known as coumarins. Numerous descriptions of their pharmacological uses highlight their uses in the treatment of various human cancers as well as in the inhibition of the growth of various cancer cell lines, including lung cancer. The capacity to inhibit many proteins associated with the illness and their low toxicity make them interesting candidates for the development of novel anti‐lung cancer medicines (Kumar et al. 2018).

Based on the medicinal potential of coumarin which has anticancer activity new derivatives of coumarins were synthesized. Their biological activities were accessed by the researcher. Nuclear magnetic resonance data was used to confirm the structures of the newly created coumarin derivatives, which were produced from 4‐hydroxycoumarin. The anti‐metastatic potential of the 10 of the produced compounds against lung cancer was studied. A number of the examined substances exhibited an inhibitory effect on the activity or movement of lung cancer cells. The usage of these substances did not result in any cytotoxic effects. The activity of the epithelial‐mesenchymal transition markers was expressed by the 4‐hydroxycoumarin derivatives which had a strong inhibitory effect on lung cancer cell movement or motility (Zhou, Kusaka, et al. 2022; Zhou, Yu, et al. 2022).

Different concentrations of the coumarin‐copper drug were administered to LA795 lung adenocarcinoma cells in order to investigate the effects of the copper complex, also known as coumarin copper drug, which contains phenanthroline and a coumarin derivative, on lung adenocarcinoma cells both in vitro and in vivo, as well as the mechanism of action. Western blot analysis was used to evaluate the expression level of apoptosis‐associated proteins, and Annexin V/propidium iodide staining was used to identify cell apoptosis using flow cytometric analysis. The cell proliferation was calculated using the MTT assays (the gold standard for the determination of cell viability and cell proliferation). Additionally, a mouse model of LA795 cells xenograft tumor was formed by injecting either phosphate‐buffered saline or coumarin‐copper medication intraperitoneally once a week for 3 weeks at a dose of two or four milligrams per kilogram. Tumor growth inhibition rates were computed and tumor growth curves were plotted. Additionally, the apoptotic index revealed that the coumarin copper medication greatly expressed the growth of LA795 tumors in a dose‐dependent manner. Finally, by inducing cell death, the coumarin copper medication may prevent the growth of LA795 cells (Zhu et al. 2018).

## 13 Stomach Cancer

Often called stomach cancer, this disease is the fifth most frequent type of cancer worldwide and ranks third in terms of cancer‐related mortality. The onset of stomach cancer may be caused by a variety of reasons. Advanced age, male sex, ethnicity, and genetic variables may have a role in the development of stomach cancer. However, *H. pylori* infection, dietary variables, and behavioral factors including alcohol consumption and cigarette smoking also have a role in the occurrence of stomach cancer (Poorolajal et al. 2020). The several physiologically active components of coumarin play an important role in the drug discovery process. More attention has recently been paid to coumarin derivatives as a potential cancer treatment. Using in vivo and in silico methods, researchers try to investigate how styrene‐substituted biscoumarin (SSBC) affects cancer proliferation and induces apoptosis. Subsequently, to compare its anti‐proliferative efficacy on stomach cancer cell line AGS, an MTT experiment was performed. During molecular docking, an interaction study was carried out between the most active SSBC molecule and anti‐apoptotic protein (BCL2). Two forms of lung normal cell lines, L‐132 and MRC‐5 and AGS cell lines, were employed in the determination of the SSBC inhibitory concentration (IC50). An in silico study also shows that SSBC may interact with the BH3 domain active site which may trigger apoptotic‐mediated cell death by the protein.

According to research, SSBC may be highly useful against AGS by triggering apoptosis through the intrinsic mechanism (Perumalsamy et al. 2018).

## 14 Blood Cancer

Leukemia is a kind of cancer that impacts the body's lymphatic framework and bone marrow systems, which produce blood. Leukemia is categorized as either acute or chronic based on how the tumor appears under a microscope as well as how it spreads and develops. Acute leukemia typically affects youngsters but chronic leukemia usually affects adults. Different blood cancers can be classified based on the type of cells and the duration of an illness. The prognosis for certain blood cancer types is worse and occurs sooner. Compared to other malignancies, leukemia is more common among children, accounting for 30%–35% of all cancers during this time (Akkol et al. 2020).

There is an urgent need to find new and innovative anticancer drugs due to the restricted pharmacological alternatives and the severe side effects connected to the available treatments. In light of this, the anticancer potential of marmesin, a naturally occurring coumarin, has been investigated for its anticancer potential in human leukemia cell lines and healthy human monocytes. Marmesin was shown to act cytotoxically in a dose‐dependent way. However, the IC‐50 values of 125 micro/meter indicated that the cytotoxic effects of marmesin were relatively lower for normal human monocytes. Marmesin dose‐dependently causes apoptosis and blocks cell‐colony formation. Marmesin also considerably reduced the leukemia cell's ability to migrate and caused cell cycle arrest surprisingly, at the dose of 30 mg/kg in vivo, marmesin greatly inhibits the growth of tumors. It identified that marmesin might work as a novel anticancer therapeutic drug for leukemia treatment (Dong et al. 2017).

Under light stimulation, photo‐triggered drug delivery systems enable the controlled administration of medications loaded on photoactive platforms to the intended area. Using an in vitro model of leukemia, the synthesis and effectiveness of carbazole‐coumarin were examined. In addition, carbazol‐coumarin heterocycles are used for the photo‐controlled release of chlorambucil from PTDDs. 4‐Hydroxy carbazole was employed as the building block to create CC (carbazol coumarin)‐fused heterocycles and additional alteration of these heterocycles produced two carbazole‐coumarin. The findings show that in the in vitro cancer cell model, visible light at 405 nm causes the CC (carbazole coumarin) drug combination to undergo photolysis which effectively delivers the medication. Additionally, the fact that the IC50 of carbazole coumarin against leukemia cells is lower indicates that the TPP ligand is effective in raising the bioavailability of CC‐drugs conjugates in the treatment of leukemia (Wang et al. 2020).

The researcher separated eight coumarins from *Rhizopura mucronata* leaves. Then, using IC‐50 values of 12.9, 2.6, and 3.64 for calocoumarin B, methoxyinophyllum P, and calophyllolide against Hela cells (cervical cancer) and HL‐60 (promyelocytic leukemia cells), it was stated that these compounds had anticancer properties. Additionally, HL‐60 and K‐562 cells (chronic myelogenous leukemia cells) revealed that three hemiterpenes that were isolated from *Artemisia armeniaca* L. were toxic to have deadly effects. In the investigation, Armenia had the greatest lethal effect via cell cycle arrest, with IC‐50 values for K562, and HL‐60 of 22.5 and 71.1 micro/meter, respectively (Taniguchi et al. 2018). The primary reason for treatment failure and cancer patient death is thought to be the insensitivity of cancer cells to therapeutic medicines. The occurrence of multidrug resistance in cancer patients which is characterized by abnormally high production of transport proteins is a particularly significant issue. Many coumarin derivatives play a crucial role in the treatment of leukemia; it indicated that furanocoumarin derivatives have anticancer potential that causes human leukemia cells with resistant characteristics to undergo apoptosis (Kubrak et al. 2019).

## 15 Cervical Cancer

Currently, cervical cancer is the fourth most common type of cancer in women, not the seventh. Most cases occur in low‐income countries, making it a major concern in those areas. Every year, around 500,000 new cases are estimated to occur (Harper et al. 2022). Several novel furoxan‐based coumarin derivatives were synthesized and tested for their anti‐proliferative properties to discover new medications. Every substance showed a stronger inhibitory effect on human cervical carcinoma, compared with coumarin‐3 carboxylic acid. Thus, the new furoxan‐based coumarin structure might be a novel cornerstone for developing new anticancer drugs to treat human cancer cells (Zhang et al. 2018).

Natural substances called coumarin present anticancer effects against a variety of malignancies, and implicate that it should be formed as a coup in constructing anticancer medication. Based on the MTT assay, researchers sought to ascertain the anticancer effect of 18 O‐prenylated coumarin derivatives against cervical cancer cells and normal cells, as well as the structure–activity connections between them. Additionally, flow cytometry was utilized in the investigation of the mechanism of cell death brought on by these substances. These substances can be regarded as promising anticancer drugs for additional pre‐clinical research because they were non‐toxic to normal cells and had cytotoxic effects through apoptosis (Maleki et al. 2020). Calocoumarin B, methoxyinophyllum P, and calophyllolide extracted from *R. mucronata* leaves showed anticancer activity against cervical cancer cells (HeLa cells) with IC50 equal to 3.8, 29.9, and 36.4 μM, according to the investigation (Taniguchi et al. 2018).

A set of solid‐state emitters based on oxazolone–coumarin triazoles that can cause the human cervical cancer line HeLa to undergo apoptosis was presented. The synthesis of these novel fluorescent inhibitors involved the successive use of multi‐component reactions with oxazolone alkynes and coumarin azides. The genesis of these compounds' solid‐state emission capabilities was linked to an atypical AIE (Aggregation‐Induced Emission) process. Studies on the cytotoxicity of these compounds revealed their potential as HeLa (human cervical cancer) inhibitors. All things considered, these novel solid‐state emitters' optical and biological characteristics hold great promise for a variety of uses in science and medicine (Shamsiya and Bahulayan 2022).

## 16 Skin Cancer

Cancerous melanoma is the third most prevalent type of skin cancer, but it is also the one with the greatest death rate and increasing frequency worldwide. It is projected that 1 in 75 individuals born in 2000 will get malignant melanoma at some point in their lives, and 20% of those individuals will expire from a common illness within 5 years of receiving a diagnosis (Akkol et al. 2020). It is generally known that furocoumarins are readily absorbed from food and that they spread quickly into a variety of tissues including the skin. Furocoumarin may get photo‐activated in human skin when exposed to UV light and create interesting crosslinks with DNA. It has been used as a topical or medicine in conjunction with phototherapy to treat a variety of problems because of this feature (Melough and Chun 2018). The connection between furo‐coumarin and skin cancer has been studied in research. The two prospective cohort studies included 75,291 women from the Nurses' Health Study (NHS) and 47,453 men from the Health Professionals Follow‐Up Study (HPFS), whose dietary data were collected every 2–4 years. A furo‐coumarin meal composition was used to calculate the average and energy‐adjusted furo‐coumarin intake of the participants. Consumption of total furo‐coumarins did not significantly increase the incidence of melanoma (Sun et al. 2020).

## 17 Brain Cancer

The most deadly and invasive kind of cancer that affects the central nervous system (CNS) is brain cancer. A diverse collection of primary as well as metastatic brain cancers is referred to as brain cancer (Rabha et al. 2021). Two types of brain cancers are distinguished: primary brain cancer, also known as gliomas, spreads from its original location outside the CNS and is thought to have originated from systemic neoplasms. It then progresses to the interior of the brain parenchyma. Among the gliomas that originate from glial cells are oligodendroglioma, schwannomas, glioblastoma, and astrocytoma (Louis et al. 2021). More than one‐third of brain tumors are primary malignant tumors, which have a high morbidity and death rate (Voisin et al. 2021). Many plants naturally contain coumarin, a chemical that has been investigated for possible anticancer effects, including activity against brain tumors.

Studies have been conducted that deal with how coumarin exerts its anti‐tumorigenic effect, primarily focused on glioblastoma multiforme (GBM), one of the most common and deadliest types of brain tumor. The anticancer effect of coumarin on glioblastoma cells was a subject investigated in published work. The scientists observed that coumarin induced glioblastoma cells to undergo apoptosis (programmed cell death) and also inhibited their proliferation. They also showed that coumarin was able to block the invasion and migration, two steps in tumorigenesis, as well as cancer local invasiveness (Vakili‐Azghandi et al. 2024). The researchers synthesized several coumarin derivatives and tested their impact on glioblastoma cells. Several coumarin compounds displayed robust efficacy in inducing apoptosis and inhibiting the proliferation of glioblastoma cells. Coumarin may also be a potential therapeutic as migraines and brain tumors are fetal conditions, so finding a better mechanism is necessary (Santha and Dwivedi 2015).

## 18 Bone Cancer

The bone ranks among the most common sites of metastasis from various types of cancer. Patients with bone metastasis will have serious skeletal‐related events (SERs), and such events can largely affect the life quality of patients. These SREs are pathological fractures, pain, hypercalcemia, and spinal cord compression. In addition, the morbidity and mortality of bone metastasis have increased significantly (Huang et al. 2020). Numerous studies have involved the mechanism of anti‐tumor action expressed by coumarins in bone cancer. Coumarin has been shown to inhibit the ability of bone cancer cells to grow, proliferate, and spread through multiple mechanisms (Wu et al. 2020). It also modulates the expression of apoptotic proteins in bone cancer. Osthol is a natural coumarin derivative. It was reported to have significant inhibitory effects on alkaline transferase level and caspase‐3 activation in the treatment of bone cancer with osthol, bioconversion by replacing its methoxy group available at position 7 with three‐methyl two‐butenyl group available at position eight rather than just skullcap arises (Akkol et al. 2020). Table 1 presents the published data on coumarin against cancers.

**TABLE 1:** Coumarin against cancers.

Type of cancer	In vitro/In vivo	Cell lines	Source/Intervention	Effect/Mechanism	Reference
Breast cancer	In vivo	SKBR‐3 cells	Coumarin	Enhanced expression of p21 protein and cell cycle arrest at G0/G1 causing cell death	Küpeli Akkol et al. (2020)
Breast cancer	In vitro	MCF‐7	Coumarin	IC50 values ranged from 1.24 to 8.68 μM, indicating significant cytotoxic action against MCF‐7 cells	Ahmed et al. (2020)
Breast cancer	In vitro	MCF‐7 and MDA‐MB‐231	Coumarin	Pharmacological action has been significantly increased by 15 times	Gkionis et al. (2021)
Prostate cancer	In vivo	PC‐3 cell lines	Coumarin	Coumarin employed strong 15‐LOX‐1 inhibitory action	Maleki et al. (2023)
Prostate cancer	In vitro	PC‐3 cell lines	Coumarin	The cytotoxic activity was mild, with IC50 value of 30 μg/mL against PC‐3	Iranshahy et al. (2019)
Prostate cancer	In vitro	PC‐3 cell lines	Coumarin	Significant dose‐dependent cytotoxic impacts in the prostate cancer cell lines	Shahzadi et al. (2020)
Cervical cancer	In vitro	HeLa cells	Coumarin	HeLa cells undergo apoptosis and cell cycle arrest at G1 phase	Maleki et al. (2020)
Cervical cancer	In vitro	HeLa cells	Coumarin	Increased levels of P21 and P27, caused apoptosis, and activated caspases‐9 and 3, causing cell cycle arrest during G0/G1 phase	Abd El‐Karim et al. (2019)
Hepatic cancer	In vitro	HepG2	Coumarin	Cell growth was 50% inhibited after 72 h of incubation, compared to untreated controls	Sabt et al. (2018)
Hepatic cancer	In vivo	HepG2	Coumarin	Excellent anticancer activity by regulating genes involved in apoptosis, tumor formation and cell cycle arrest	Nasr et al. (2018)
Colon cancer	In vitro	HCT116 and SW480 cells	Coumarin	Coumarin increased cancer cell death by activating the apoptosis pathway	Lin et al. (2019)
Colon cancer	In vitro	HT‐29 cell lines	Coumarin	Autophagy through PI3K/Akt/mTOR and signaling at AMPK/mTOR on HT‐29 cells	Hoveizi and Hushmandi (2024)
Colon cancer	In vitro	HCT‐116 cell lines	Coumarin	The cytotoxic activity was measured with IC50 value of 1.93 μM for HCT‐116	Imran, Bawadekji, and Nayeem (2019)
Lung cancer	In vitro	A549 and H2170 cell lines	Coumarin	Reversing EMT in IL‐1β‐stimulated A549 cells and suppressing EMT‐associated migration in A549 cells	de Araújo et al. (2022)
Lung cancer	In vitro	A549 and H1299 cell lines	Coumarin	Compound 8b induced autophagy in H1299 and A549 cells	Wang et al. (2018)
Gastric cancer	In vitro	AGS, L‐132 and MRC‐5 cells	Coumarin	The IC50 value of SSBC for AGS, L‐132, and MRC‐5 was 4.56, 268, and 285 μg/mL, respectively	Perumalsamy et al. (2018)
Gastric cancer	In vivo	MGC‐803 and HGC‐27 cells	Coumarin	Inhibition of MAPK signaling and tumor development in MGC‐803 xenograft models	Song et al. (2022)
Ovarian cancer	In vitro	A2780 cell lines	Coumarin	Significant cytotoxicity at 100 μM in the cell lines	Karataş et al. (2019)
Ovarian cancer	In vitro	SKVCR and SV40 cell lines	Coumarin	Cell growth decreased due to apoptosis triggered by the caspase‐linked pathway	Wang and Wang (2021)
Neuroblastoma	In vitro	N2A cell line	Coumarin	Apoptosis was induced in N2A cell line	Sargolzaei et al. (2020)
Neuroblastoma	In vitro	SH‐SY5Y cell line	Coumarin	Reduced proliferation of the SH‐SY5Y cells, which induces apoptosis and increases cell population in the sub‐G0/G1 phase	Maugeri et al. (2021)

## 19 Conclusion

The molecule coumarin which is naturally occurring in a large number of plants has shown to have vast potential in being an anticancer drug due to the multifaceted molecular mechanisms. Coumarin and its derivatives’ anticancer properties have been the subject of many studies in recent years because of the numerous ways they have been found to act and the promising treatment outcomes that have been observed. Coumarins possess a benzopyrone structure, which has many biological activities, and anticancer activity that occurs through multiple pathways and targets. Anticancer activity of coumarin and its derivatives may result from its ability to reverse MDR, inhibit angiogenesis, induce oxidative stress, alter key signaling pathways, and inhibit cell cycle progression. Numerous key pathways linked to the development of cancer, including the PI3K/Akt/mTOR signaling pathway, which is necessary for cell proliferation and survival, are inhibited by coumarin. Because it can trigger apoptosis in cancer cells by altering the process, coumarin has an impact on the viability of malignancies. It also inhibits carbonic anhydrase which promotes tumor growth and acidity of tumors. The results of the various studies show that the presence of a wide variety of coumarin derivatives determines the effectiveness of their anticancer action. Several hybrids were recently synthesized that demonstrated significant cytotoxicity against breast cancer cells, lung cancer cells, and colon cancer cells. For instance, it has been shown that coumarin‐acrolein hybrids not only inhibit cell proliferation but do this with relative sparing of normal cells. Definite receptors in cancerous cell subtypes have been developed by coumarin modification and reducing side effects on healthy tissues enhancing its therapeutic efficacy. The prospects for coumarin‐based medicines in the future are promising. Structure–activity (SAR) analysis is being carried out to improve the pharmacological properties of coumarin derivatives. These studies focus on enhancing the efficacy of the intervention and, at the same time, decreasing the toxicity of the process. These drugs' solubility and accumulation at tumor sites can also be improved by advanced drug delivery systems. In addition, the incorporation of coumarins into combination therapies may enhance the efficacy of the drugs in treating cancer types that are unresponsive to such treatments due to the issue of multidrug resistance evident in many cancers.

## Author Contributions

**Muhammad Shahbaz:** data curation (equal), writing – original draft (equal). **Asfa Perween:** data curation (equal), writing – original draft (equal). **Ushna Momal:** methodology (equal), writing – review and editing (equal). **Muhammad Imran:** conceptualization (equal), validation (equal), visualization (equal). **Muhammad Hammad Ul Hassan:** data curation (equal), validation (equal). **Hammad Naeem:** conceptualization (equal), writing – review and editing (equal). **Ahmed Mujtaba:** data curation (equal), investigation (equal), resources (equal). **Muzzamal Hussain:** writing – review and editing (equal). **Suliman A. Alsagaby:** investigation (equal), writing – review and editing (equal). **Waleed Al Abdulmonem:** data curation (equal), visualization (equal). **Mohamed A. Abdelgawad:** conceptualization (equal), writing – review and editing (equal). **Ahmed H. El‐Ghorab:** conceptualization (equal), investigation (equal), supervision (equal). **Samy Selim:** conceptualization (equal), resources (equal), supervision (equal). **Ehab M. Mostafa:** investigation (equal), software (equal), visualization (equal). **Entessar Al Jbawi:** data curation (equal), supervision (equal).

## Consent

The authors have nothing to report.

## Conflicts of Interest

The authors declare no conflicts of interest.

## Data Availability

The authors confirm that the data supporting the findings of this study are available within the article.
